# BRCC3 acts as a prognostic marker in nasopharyngeal carcinoma patients treated with radiotherapy and mediates radiation resistance in vitro

**DOI:** 10.1186/s13014-015-0427-3

**Published:** 2015-05-30

**Authors:** Ziwei Tu, Bingqing Xu, Chen Qu, Yalan Tao, Chen Chen, Wenfeng Hua, Guokai Feng, Hui Chang, Zhigang Liu, Guo Li, Changbin Jiang, Wei Yi, Musheng Zeng, Yunfei Xia

**Affiliations:** Department of Radiation Oncology, Sun Yat-Sen University Cancer Center, No. 651, Dongfeng East Road, Guangzhou, 510060 People’s Republic of China; State Key Laboratory of Oncology in South China, Sun Yat-Sen University Cancer Center, Guangzhou, Guangdong, People’s Republic of China; Department of Radiation Oncology, Hunan Cancer Hospital, The Affiliated Cancer Hospital of Xiangya School of Medicine, Central South University, Changsha, Hunan People’s Republic of China; Department of Radiation Oncology, Affiliated Tumor Hospital, Guangzhou Medical University, Guangzhou, Guangdong People’s Republic of China; Department of Radiation Oncology, The First Affiliated Hospital of Guangzhou Medical University, Guangzhou, People’s Republic of China

**Keywords:** BRCC3, Nasopharyngeal carcinoma, Prognostic marker, Radioresistance

## Abstract

**Background:**

BRCC3 has been found to be aberrantly expressed in breast tumors and involved in DNA damage response. The contribution of BRCC3 to nasopharyngeal carcinoma prognosis and radiosensitivity is still unclear.

**Methods:**

Immunohistochemical analysis of BRCC3 was carried out in 100 nasopharyngeal carcinoma tissues, and the protein level was correlated to patient survival. BRCC3 expression of nasopharyngeal carcinoma cell lines was determined by Western-blotting and real-time PCR. Additionally, the effects of BRCC3 knockdown on nasopharyngeal carcinoma cell clongenic survival, DNA damage repair, and cell cycle distribution after irradiation was assessed.

**Results:**

The BRCC3 protein level was inversely correlated with nasopharyngeal carcinoma patient overall survival (*P* < 0.001) and 3-year loco-regional relapse-free survival (*P* = 0.034). Multivariate analysis demonstrated that BRCC3 expression was an independent prognostic factor (*P* = 0.010). The expression of BRCC3 was much higher in radioresistant nasopharyngeal carcinoma cells than in radiosensitive cells. Knockdown of BRCC3 increased the cell survival fraction, attenuated DNA damage repair and resulted in G2/M cell cycle arrest in radioresistant NPC cells.

**Conclusions:**

High BRCC3 expression in nasopharyngeal carcinoma patients is associated with poor survival. BRCC3 knockdown could abate the radioresistance in nasopharyngeal carcinoma cells. These findings suggest the utility of BRCC3 as a prognostic biomarker and novel target for nasopharyngeal carcinoma.

## Background

Nasopharyngeal carcinoma (NPC) is a form of epithelial cancer with high occurrence rates in Southeast Asia and southern China, where its incidence is approximately 25 to 50 cases per 100,000 individuals [1]. Radiotherapy has been used as the primary treatment of nasopharyngeal carcinoma [2], and a majority of NPC patients can be cured when diagnosed and treated at an early disease stage. However, approximately 20 % of NPC patients suffer from local recurrence after treatment [3], and radiosensitivity is widely perceived as one of the major obstacles for radiotherapy.

Investigators have made many efforts to understand the DNA damage response(DDR). In this process, factors gathered to sites of DNA damage within minutes of irradiation [4–6], and initiated a phosphorylation signaling cascade. Firstly, DNA damage induce ATM/ATR phosphorylation on S139 of histone H2AX, then directly recruits MDC1 through its BRCT domains, and the phosphorylation of MDC1 leads to the recruitment of an ubiquitin ligase RNF8/UBC13 to damage sites. The subsequent ubiqutination events on the damaged chromatin create docking sites for DNA repair proteins to accumulate at DNA double strand breaks(DSBs) [7–11]. Following the resolution of DNA damage, repair proteins dissociate from DSBs, thus alleviating cell cycle checkpoint responses and allowing resumption of cell proliferation.

BRCA1-BRCA2-containing complex(BRCC), a novel multiprotein complex composed of BRCA1, BARD1, BRCA2, RAD51, BRCC3 and BRCC45 in addition to other proteins [12], was reported to participate in the DNA damage reponse illustrated above [13–15]. As one of the subunits of BRCC, BRCC3 functions to counteract Ubc13-RNF8 activity to provide a balanced level of ubiquitin near DNA lesions, which is essential for the recruitment and dissociation of DNA repair proteins [15, 16]. Knocking down BRCC3 expression impairs the DNA repair pathway [15, 17], resulting in the disruption of G2/M checkpoint arrest [12] and increased cell apoptosis [17]. Furthermore, BRCC3 is overexpressed in the vast majority of breast tumors [12]. Taken together, this suggests that BRCC3 is accountable for cell radioresistance and has potential clinical relevance in breast cancer. Thus, we hypothesize that BRCC3 plays a similar role in NPC radioresistance and accounts for the poor prognosis of NPC patients.

To study the clinical application value of BRCC3, we determined the relationship between the BRCC3 expression level and nasopharyngeal carcinoma patient survival. Moreover, we investigated the contribution of BRCC3 to radiation resistance in HNE1 and CNE2R cells, two human nasopharyngeal carcinoma cell lines that expressed a high level of BRCC3 and exhibited resistance to radiation.

## Methods

### Immunohistochemical staining (IHC)

#### Tissue samples

This study was conducted on a total of 100 paraffin-embedded NPC tissue samples obtained from patients who were histologically and clinically diagnosed at the Sun Yat-Sen University Cancer Center, China, between 1994 and 1999. Patient consent and approval from the Institute Research Ethics Committee was obtained prior to the use of these clinical materials for research purposes. The clinical characteristics of the patients are shown in Table 1.

**Table 1:** Correlation between the clinicopathologic features and expression of BRCC3

		BRCC3			
Characteristics	N	Low expression	High expression	*χ* ^2^	*P* values
Gender					
Male	77	41	36	1.030	0.310^a^
Female	23	15	8		
Age					
<45	47	27	20	0.075	0.784 ^a^
≥45	53	29	24		
Histological classification					
Type II	8	5	3	0.149	0.699 ^b^
Type III	92	51	41		
Clinical stage					
I-II	56	25	31	6.662	0.010 ^a ^*
III-IV	44	31	13		
T					
T1-T2	66	39	27	0.753	0.386 ^a^
T3-T4	34	17	17		
N					
N0	60	39	21	4.931	0.026 ^a ^*
N1-N3	40	17	23		
M					
M0	83	52	31	8.764	0.003 ^a^ *
M1	17	4	13		
Relapse					
Yes	14	8	6	0.009	0.926 ^a^
No	86	48	38		
Skull-based invasion					
Yes	20	11	9	0.010	0.920 ^a^
No	80	45	35		

^a^ Pearson Chi-Square test (asymptotic significance, two-sided)

^b^ Fisher’s exact test (two-sided)

* Significantly different

#### Immunohistochemical staining (IHC)

IHC staining was performed using the Dako Envision system (Dako, Carpinteria, CA) following the manufacturer’s recommended protocols. All paraffin-embedded specimens were cut into 4-μm sections and baked for 2 h at 65 °C. All sections were deparaffinized with xylenes and rehydrated with graded ethanol to distilled water and then submerged in EDTA antigen retrieval buffer (pH 8.0) and microwaved for antigen retrieval. After being treated with 0.3 % H2O2 for 15 min and normal goat serum for 30 min, the sections were incubated with a BRCC3 antibody (1:200; Pierce Biotechnology; PA5-20426) overnight at 4 °C. After washing, the sections were incubated with a biotinylated anti-rabbit secondary antibody (Zymed) followed by further incubation with streptavidin-horseradish peroxidase (Zymed) at 37 °C for 30 min. For the color reaction, diaminobenzidine (DAB) was used.

We used the intensity and extent of the staining to assess BRCC3. The entire tissue section was observed to assign scores. The staining intensity was scored as 0 (no staining), 1 (weak staining exhibited as light yellow), 2 (moderate staining exhibited as yellow brown), or 3 (strong staining exhibited as brown). The extent of staining was scored as 0 (0 %), 1 (1 to 25 %), 2 (26 to 50 %), 3 (51 to75%), or 4 (76 to 100 %), according to the percentages of the positive staining areas in relation to the whole carcinoma area or the entire section for the normal samples. The product of the intensity and extent scores was used as the final staining score (0 to 12) for BRCC3. For the purpose of statistical evaluation, tumors having a final staining score of <4 were grouped into low BRCC3 expression, and those with scores ≥4 were grouped into high BRCC3 expression [18].

### Cell culture, siRNA transfection, and IR

We received the identified CNE2 and CNE2R cells (tested by analysis of the DNA microsatellite short tandem repeats) from Professor Hui-ling Yang (Department of Pathophysiology, Zhongshan School of Medicine, Sun Yat-Sen University) [19, 20]. Other human NPC cell lines (CNE1, SUNE1, SUNE2, HNE1, HONE1) were maintained in Sun Yat-Sen University Cancer Center [21, 22] and were not authenticated prior to this study. All of the NPC cell lines were maintained in RPMI 1640 (Invitrogen, Carlsbad, CA) supplemented with 10 % fetal bovine serum (FBS; Hyclone, Logan, UT), penicillin (100 units/ml), and streptomycin (100 units/ml) at 37 °C in a humidified 5 % CO2 incubator.

For the BRCC3 depletion studies, NPC cells were plated at a density of 3 × 10^5^ cells/cm^2^. After reaching 30 % to 40 % confluence, cells were transfected with siRNA using riboFECT™ CP and OPTI-MEM I reduced serum medium (Invitrogen/Life Technologies, Inc., Carlsbad, CA) according to the manufacturer’s protocol. The siRNA duplexes with the following sense and antisense sequences were used, siRNA1: 5′-GAGGAAGGACCGAGUAGAAdTdT (sense) and 5′-UUCUACUCG-GUCCUUCCUCdTdT (antisense); siRNA2: 5′-AACAUCAACAUGUGAAGGCdTdT(sense) and 5′-GCCUUCACAUGUUGAUGUUdTdT(antisense) [13, 17]. All of the siRNA duplexes were synthesized by RIBOBIO. Twenty-four hours after transfection, the cells were irradiated using a R2000 X-ray irradiator (1.1 Gy/min, 160 kV, 25 mA, 0.3 mm copper filters).

### Colony-forming assay

Cells were transfected with the indicated siRNAs, and 24 h later, they were trypsinized, seeded into 6-well plates at different densities(100, 200, 10^3^, 10^4^cells for 0Gy, 2Gy, 4Gy, 6Gy groups separately) and X-ray irradiated at defined doses. After 7–14 days of incubation, the cultures were fixed and stained with Giemsa. Colonies with more than 50 cells were scored as survivors. The plating efficiency (PE) was calculated by dividing the number of colonies counted by the number of cells plated. The surviving fractions (SF) were then calculated by dividing the PE by the PE of the non-irradiated control [15, 23, 24]. Sensitization enhancement ratio(SER) was calculated by division of SF2(surviving fractions at 2Gy). Clonogenic survival curves were compared through the extra sum-of-squares F test in GraphPad Prism [25].

### Real-time RT-PCR

Total RNA from different NPC cell lines were extracted with Trizol reagent (Invitrogen, Carlsbad,CA), then the first-strand complementary DNA (cDNA) was synthesized with 1 μg of total RNA. Realtime-PCR was performed in triplicate by using the Absolute blue SYBER green Rox mix (Thermo Scientific). PCR reaction and data collection were conducted with an ABI PRISM 7900HT sequence detection system For normalization, GAPDH was used as endogenous control. The primer sequences are sense 5′-AATTTCTCCAGAGCAGCTGTCTG, anti-sense 5′-CATGGC TTGTGTGCGAACAT for BRCC3, and sense 5′-CTCCTCCTGTTCGACAGTCAG C-3′, Antisense 5′- CCCAATACGACCAAATCCGTT-3′ for GAPDH.

### Western blot analysis

Western blot analysis was performed as described previously [18]. Briefly, total cellular proteins were extracted with lysis buffer. The protein concentration was determined by BCA Protein Assay Kit (Beyotime Biotechnology). Equal amounts of protein samples were loaded and separated by SDS-PAGE gels, electrophoretically transferred to polyvinylidene difluoride membranes(Millipore). The membrane was probed with primary antibody 4 °C overnight and corresponding secondary antibody, The enhanced chemiluminescence was conducted according to the manufacturer’s suggested protocols(Amersham Pharmacia Biotech, Piscataway, NJ). Antibodies: BRCC3 (1:1000; Pierce Biotechnology; PA5-20426), CyclinB1 (1:2000; Cell Signaling; #4135), GAPDH (1:5000; Bioworld Technology, Inc.; BSAP0063).

### Immunofluorescence analysis

Cells were transfected with the indicated siRNAs and were trypsinized 24 h after transfection, seeded on glass coverslips in a 24-well plate for 24 h, and irradiated at the indicated doses. γ-H2AX immunofluorescence analysis was carried out as described previously [24]. At 0 h, 0.5 h, 6 h, 12 h, and 24 h post irradiation, cells were fixed with 4 % paraformaldehyde (15 min, AppliChem, Darmstadt, Germany) at room temperature (RT) and permeabilized by the addition of 0.25 % Triton-X 100 in PBS for 15 min, followed by blocking in 5 % bovine serum albumin (BSA) in PBS for 30 min. Next, cells were incubated with Phospho-Histone H2AX (Ser139) (20E3) rabbit mAb (1:1000; Cell Signal Technology; #9718S), followed by the appropriate Alexa 488-conjugated (green; Molecular Probes) secondary antibodies Subsequently, nuclei were counterstained with DAPI solution (Invitrogen) and coverslips were mounted with Vectashield (Vector Laboratories, Peterborough, UK). Images were taken using an Olympus confocal imaging system (Olympus FV100) for the quantification of γH2AX foci formation. At least three independent experiments were performed for each data point.

### Cell cycle analysis

Cell cycle analysis was performed as described previously [26]. Briefly, cells were harvested 24 h post-radiation by trypsinization and washed with phosphate-buffered saline (PBS). For cell cycle analysis, the cells were fixed with 70 % ethanol at -20 °C overnight. On the following day, the fixed cells were washed with PBS, treated with RNase A (50 μg/ml) in PBS at 37 °C for 20 min, and then mixed with propidium iodide (PI, 50 μg/ml) for 30 min in the dark. The stained cells were analyzed with fluorescence-activated cell sorting (FACS) by flow cytometry (FACSCalibur, Becton Dickinson, Bedford, MA). The cell-cycle profile was analyzed using the ModFit software (Becton Dickinson). At least 10,000 cells in each sample were analyzed to obtain a measurable signal.

### Statistical analysis

The Student *t*-test or Chi-square test was used to compare the differences as appropriate. Survival analysis was conducted by the Kaplan-Meier method and the log-rank test. Multivariate analysis with Cox proportional hazards model was employed to investigate independent prognostic factors. A *P*-value of < 0.05 was considered statistically significant. Statistical analysis was executed by the SPSS software package (version 16.0, SPSS Inc) or GraphPad prism 5.

## Results

### BRCC3 expression is inversely correlated with the survival of NPC patients

**Fig. 1:**
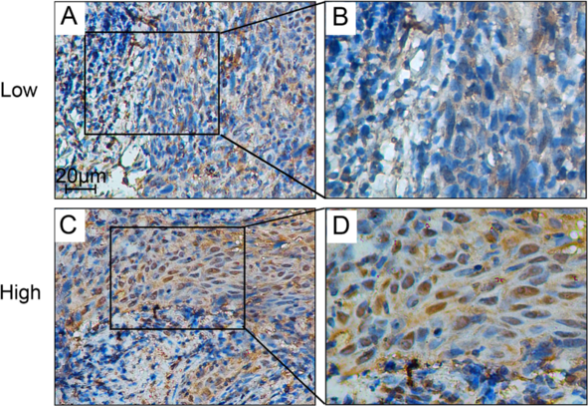
Immunohistochemistry staining of BRCC3 expression in nasopharyn-geal carcinoma tissues. BRCC3 expression was mainly localized within the nuclei of cancer cells. **a** Tumor with low BRCC3 level (200×); **c** Tumor with high BRCC3 level (200×); **b** and **d** demonstrated the higher magnification (400×) from the area of the box in (**a**) and (**c**) respectively. Low: low BRCC3 expression; High: high BRCC3 expression

**Fig. 2:**
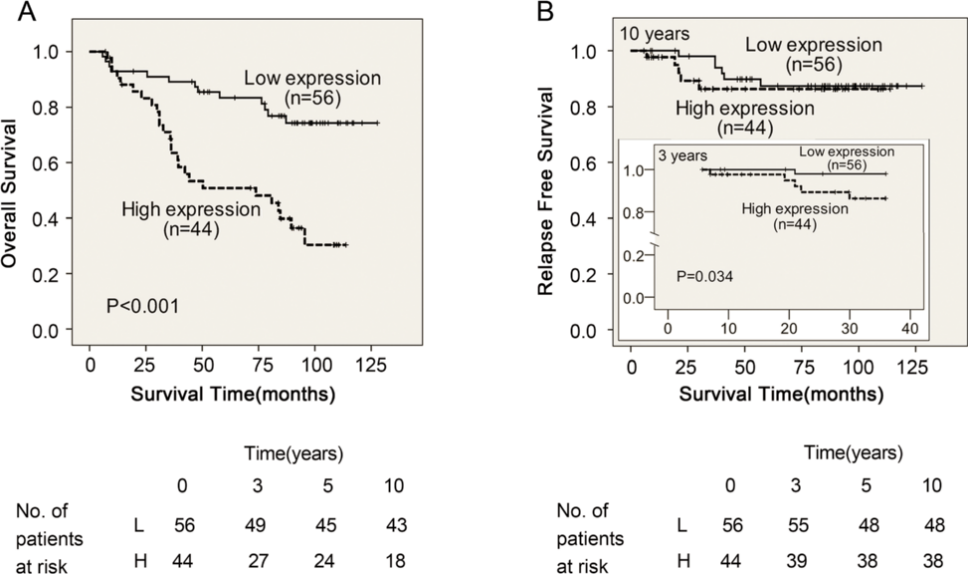
A negative correlation between BRCC3 expression and NPC patient survival. **a** Overall survival in NPC patients according to BRCC3 expression. Patients with high BRCC3 expression (*n* = 44) possessed with significantly poor overall survival compared with that of patients with low BRCC3 expression (*n* = 56) (*P* < 0.001). Number of patients at risk at 0, 3, 5, 10 years is shown below the survival curves (L, patients with low BRCC3 expression level; H, patients with high BRCC3 expression level). **b** Relapse Free Survival in NPC patients. The BRCC3 protein level was inversely correlated with nasopharyngeal carcinoma patient 3-year loco-regional relapse-free survival (*P* = 0.034)

To determine the impact of BRCC3 on survival, BRCC3 protein expression was determined by immunohistochemistry in NPC patient tissues. The expression of BRCC3 was primarily localized to the nucleus of cancer cells (Fig. 1). The correlation between BRCC3 protein levels and various clinicopathological factors are shown in Table 1. No statistically significant correlations were observed between the expression of BRCC3 and age at diagnosis, gender, tumor size, or histological classification. Statistically significant correlations between high levels of BRCC3 expression were found with the clinical stage and metastasis. Kaplan–Meier analysis revealed that patients with a high expression of BRCC3 in NPC tissues had a worse overall survival (OS) (Fig. 2a, log-rank test: *P* < 0.001) than those with low BRCC3 expression. BRCC3 expression had no significant effect on the 10-year loco-regional relapse-free survival (LRRFS) of NPC patients (Fig. 2b). Nevertheless, BRCC3 overexpression increased the risk of recurrence within three years (Fig. 2b, *P* = 0.034).

Univariate analysis indicated that the clinical stage, T classification, N classification, distant metastasis, relapse, skull-base invasion and BRCC3 expression were statistically significant prognostic factors. However, the clinical prognosis was not related to age, gender or histological classification. Multivariate analysis including the BRCC3 expression level, T classification, N classification, distant metastasis and skull-base invasion demonstrated that the BRCC3 expression level (*P* = 0.01), distant metastasis (*P* < 0.001), relapse (*P* = 0.001), and skull-base invasion (*P* = 0.013) were independent prognostic factors for NPC (Table 2). Thus, our findings indicate that the BRCC3 expression level, as an independent prognostic factor, is inversely associated with the clinical prognosis of NPC.

**Table 2:** Univariate and Multivariate analysis of factors associated with overall survival

Variables		Univariate analysis		Multivariate analysis		
	HR	95 %CI	P^a^	HR	95%CI	P^a^
Gender	0.725	0.320-1.642	0.440			
Male vs Female						
Age (years)	1.106	0.589-2.077	0.753			
<45 vs ≥ 45						
Histological classification	1.602	0.568-4.516	0.373			
Type II vs Type III						
Clinical stage	3.402	1.651-7.007	0.001			
I-II vs III-IV						
T classification	1.886	0.999-3.558	0.050			
T1-T2 vs T3-T4						
N classification	2.328	1.233-4.395	0.009			
N0 vs N1-N3						
Distant metastasis	6.223	3.091-12.529	<0.001	7.026	2.898-17.037	<0.001
No vs Yes						
Relapse	2.648	1.248-5.619	0.011	5.081	2.017-12.802	0.001
No vs Yes						
Skull-based invasion	2.483	1.226-5.029	0.012	2.996	1.258-7.133	0.013
No vs Yes						
Radiotherapy response	2.949	1.462-5.946	0.003			
Sensitive vs Resistant						
BRCC3	3.560	1.821-6.958	<0.001	2.674	1.270-5.630	0.010
Low vs High						

Abbreviation: OS, overall survival; 95 % CI, 95 % confidence interval; HR, hazard ratio

^a^. Cox proportional hazards model

### BRCC3 is over-expressed in radioresistant NPC cells

**Fig. 3:**
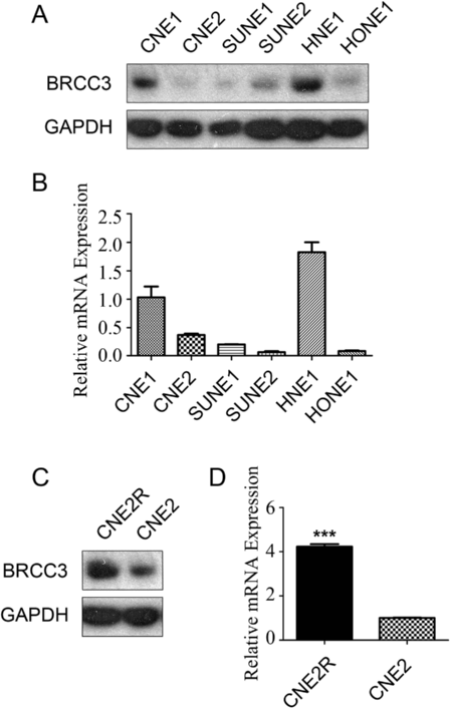
High expression level of BRCC3 in radioresistant NPC cell lines. **a** Western blot analysis of BRCC3 NPC cell lines; **b** Real-time PCR analysis of BRCC3 protein in the same cell lines as described in A. **c** Western blotting analysis of BRCC3 in CNE2 and CNE2R cells. **d** Realtime PCR analysis of BRCC3 in CNE2 and CNE2R cells. *T* test was used to compare the expression level, the asterisks indicate a significant (***p < 0.001) difference

To investigate the exact role of BRCC3 in NPC, we first determined the BRCC3 expression levels in different NPC cell lines by using Western blotting and real-time PCR (Fig. 3a and b). Interestingly, both methods revealed a relatively higher BRCC3 expression level in the radioresistant cell lines CNE1 and HNE1 [27]. Based on this, we determined the amount of BRCC3 expression in CNE2 and resistant CNE2 (CNE2R) [19, 20]. Consistent with the results above, we found a significantly upregulated level of BRCC3 expression in CNE2R cells (Fig. 3c and d). These results indicated that BRCC3 may participate in the regulation of NPC radiosensitivity.

### BRCC3 knockdown sensitizes NPC cells to ionizing radiation

**Fig. 4:**
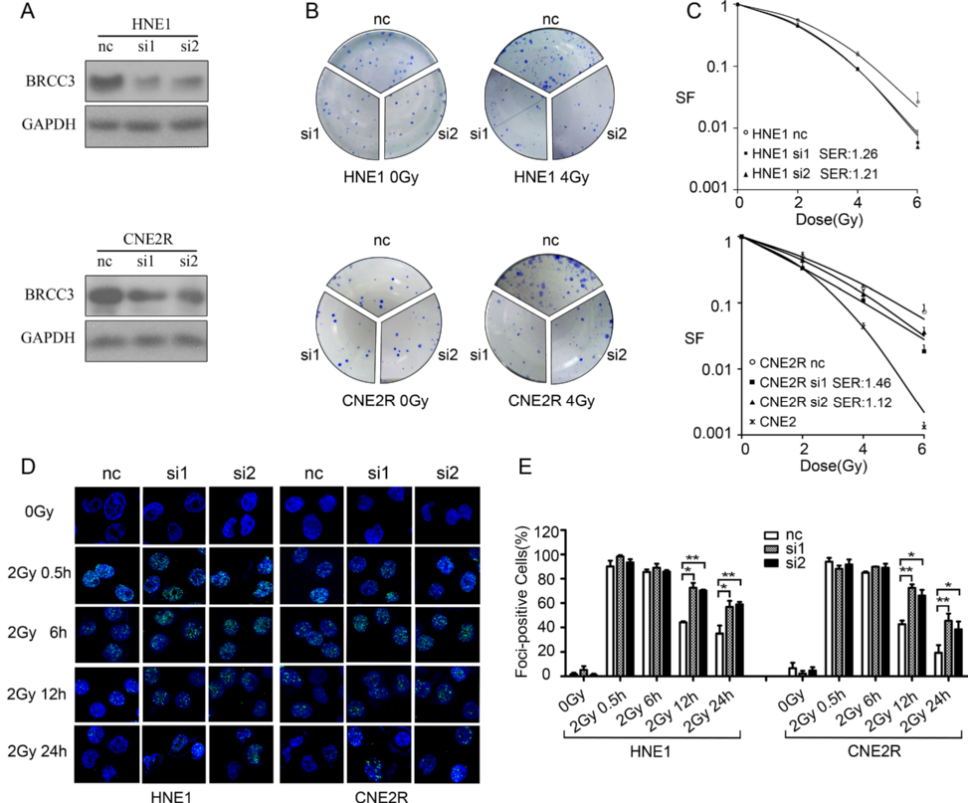
Depletion of BRCC3 results in increased sensitivity to ionizing radiation. **a** Knockdown of BRCC3 was assessed by western-blotting (nc: negative control siRNA; si1: siRNA1 target BRCC3; si2: siRNA2 target BRCC3). **b** HNE1 and CNE2R cells were transfected with siRNAs, 24 h later they were seeded to 6-well plates and irradiated with different doses. Clones were stained and counted after 7-14 days. **c** Dose-survival curves of irradiated HNE1 and CNE2R cells (range, 0-6Gy, SF: survival fractions, SER: radiation sensitization enhancement ratio). In HNE1 cells SER of si1 and si2 was 1.26 (*p* < 0.05) and 1.21 (*p* < 0.05) separately; In CNE2R cells SER of si1 and si2 was 1.46 (*p* < 0.01) and 1.12 (*p* < 0.05) separately. **d** Immunofluorescent staining of γH2AX. HNE1 and CNE2R cells transfected with BRCC3 or control siRNA was exposed to 2Gy irradiation, and immunofluorescent staining of γH2AX foci was conducted before and 0.5 h, 6 h, 12 h, 24 h after irradiation (**e**) Quantification of γH2AX foci formation. A positive cell was determined by the presence of >10 foci/cell. The percentage of positive cells was compared by *t*-test. The asterisks indicate a significant (*p < 0.05,**p < 0.01) difference

Since high level of BRCC3 was correlated with poor prognosis in NPC patients that treated by radiotherapy, we sought to explore the association between BRCC3 expression and radiation resistance in vitro. We performed in vitro silencing studies targeting BRCC3 in HNE1 and CNE2R cell lines, which express relatively high levels of BRCC3 (Fig. 3). The silencing efficiency was confirmed by western-blotting (Fig. 4a). Following transfection with control or BRCC3 siRNAs, we exposed CNE2 cells, transfected HNE1 and CNE2R cells to ionizing radiation and compared the survival fractions using colony formation assays, which revealed that CNE2R cells were more resistant than CNE2 cell, BRCC3 knockdown cells had significantly decreased survival fractions compared to the corresponding control cells. In HNE1 cells SER of siRNA1 and siRNA2 was 1.26 and 1.21 separately; In CNE2R cells SER of siRNA1 and siRNA2 was 1.46 and 1.12 separately (Fig. 4b,c).

Next, we evaluated the DNA damage response (DDR). BRCC3, as a Lys 63-linked ubiquitin-specific de-ubiquitinating enzyme (DUB), plays an important role in the DDR, such as removing unnecessary DNA repair factors after DSB repairs [15]. Therefore, to study the effect of BRCC3 knockdown on the induction and repair of DSBs in NPC cells, Cells were transfected with BRCC3 or control siRNAs 24 h prior to irradiation (2 Gy), and we conducted immunofluorescence before and 0.5 h, 6 h, 12 h, 24 h after irradiaton to examine the phosphorylation of H2A.X at Ser139 (γH2AX) DNA repair foci in response to ionizing radiation. Our results showed that the deficiency in BRCC3 did not result in a significant difference of DSB levels compared to control cells before radiation and 0.5 h after radiation. However, compared with control cells, a significantly higher pecentage of γ-H2AX positive cells was observed in BRCC3 knockdown cell lines HNE1 and CNE2R 12 h (HNE1control: 44.17 ± 0.83; HNE1 siRNA1: 72.57 ± 4.0; HNE1 siRNA2: 70.39 ± 0.70; CNE2R control: 42.65 ± 2.97; CNE2R siRNA1: 72.42 ± 3.0; CNE2R siRNA2: 66.16 ± 4.71) and 24 h (HNE1control: 35.08 ± 6.4; HNE1 siRNA1: 56.88 ± 4.97; HNE1 siRNA2: 58.95 ± 2.01; CNE2R control:19.38 ± 6.22; CNE2R siRNA1:45.40 ± 5.92; CNE2R siRNA2: 38.47 ± 6.37) after irradiation (Fig. 4d,e). These data indicated that the loss of BRCC3 did not affect the induction of γ-H2AX foci, but resulted in a significant delay of the γ-H2AX foci absorption after exposure to ionizing radiation in HNE1 or CNE2R cells, which implies the disruption of DSB repair. Thus, we concluded that BRCC3 knockdown observably sensitized NPC cell lines to irradiation.

### Depletion of BRCC3 results in G2/M cell cycle arrest

**Fig. 5:**
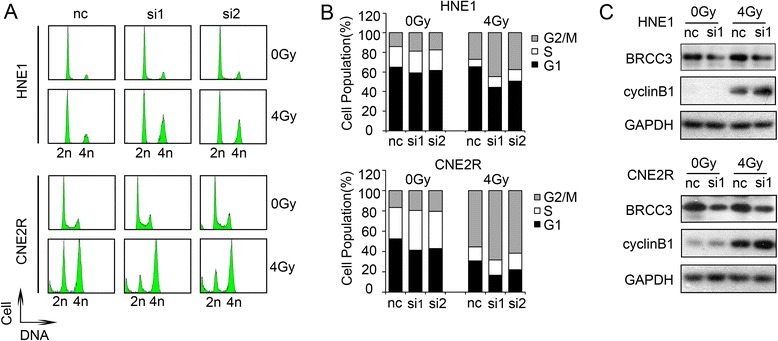
BRCC3 knockdown intensifies G2/M checkpoint arrest. **a** Cell-cycle profiles of HNE1 and CNE2R cells. Control and BRCC3 siRNA transfected cells were treated with 0Gy or 4Gy, and the FACS analysis was executed 24 h post- irradiation.(nc: negative control siRNA; si1: siRNA1 target BRCC3; si2: siRNA2 target BRCC3). **b** Quantification of A. BRCC3 inhibition caused an increased portion of G2/M phase in HNE1 and CNE2R cells. **c** Western blot analysis of cyclinB1 24 h after 0Gy or 4Gy IR

Cell cycle regulation was thought to be the foremost determinant of ionizing radiation sensitivity [28]. Thus, we used flow cytometric analysis to determine the effect of BRCC3 on cell-cycle distribution after irradiation. Control and BRCC3 siRNA transfected NPC cells were exposed to 0Gy or 4Gy iradiation, and the FACS analysis was executed 24 h post- treatment. As seen in Fig. 5a and b, radiation significantly disturbed the cell cycle progression and caused a dramatic increase of G2/M phase populations in HNE1and CNE2R cells compared with the uniradiated cells. No significant difference was noted between the BRCC3 knockdown and control cell cycle profiles in the absence of ionizing radiation. In contrast, 24 h following a 4Gy dose of ionizing radiation, a significantly greater percentage of BRCC3 knockdown cells remained in the G2/M phase compared to control cells, and correspondingly, a smaller percentage of BRCC3 knockdown cells were present in the G1 phase. This suggested that BRCC3 knockdown enhanced the G2/M cell cycle arrest induced by ionizing radiation.

We next sought to examine the expression of cell cycle-related proteins. In mammals, cell-cycle progression is dependent on highly ordered events controlled by the cyclins and cyclin-dependent kinases (Cdks) [29]. The cyclin B1/Cdk1 complex relocates in the matrix of the mitochondria, and an increased influx of mitochondrial cyclin B1/Cdk1 was associated with the elevated mitochondrial bioenergetics during the G2/M transition [30]. The results of western blotting revealed that cyclinB1 was significantly upregulated after 4Gy ionizing radiation in HNE1 and CNE2R cells and that the depletion of BRCC3 exacerbates the increase (Fig. 5c).

## Discussion

In this study, we explored the effect of BRCC3 on NPC patient survival and NPC cellular radiosensitivity. The key findings of this work are described in the following: First, it indicates a negative correlation between BRCC3 expression and NPC patient survival (Fig. 2); therefore, BRCC3 may be a potential prognostic biomarker for NPC. Second, it demonstrates that the expression of BRCC3 was upregulated in both inherent (CNE1, HNE1) and induced (CNE2R) radioresistant cell lines (Fig. 3), which suggests that BRCC3 might be a critical factor in the NPC radioresistance. Third, it reveals that knockdown of BRCC3 expression through the use of siRNAs sensitizes HNE1 and CNE2R cells to ionizing radiation, impairs γH2AX foci absorption and induces G2/M cell cycle arrest (Figs. 4 and 5), supporting a role for BRCC3 in cellular responsiveness to ionizing radiation, DNA repair and G2/M checkpoint progression.

In addition to the effect of BRCC3 on patient overall survival, interestingly, the present study discovered an unforeseen correlation between BRCC3 expression and metastasis (Table 1). A previous study demonstrated that BRCC3 gene knockdown caused a decline in the migration and invasion capabilities of glioma cells [31]. Furthermore, high expression of DNA repair pathways was associated with metastasis in melanoma patients, and BRCA1 showed a higher expression level in metastatic patients [32]. Accordingly, in bladder carcinoma [33] and breast cancer [34, 35] studies, the majority of the significant repair genes were overexpressed in the primary tumors that are going to metastasize. The proportion of significant repair genes that are overexpressed in cancers that will metastasize reaches 90 % in melanomas, 82 % in bladder carcinomas, 80 % in the van’t Veer breast cancer and 58 % in the breast cancers from Wang’s study [35]. Consequently, the correlation between BRCC3 expression and metastasis in NPC patients is not surprising. The current study revealed the relationship between BRCC3 and NPC metastasis. Further research is necessary to determine the correlation.

In addition to the pertinent literature indicating the involvement of BRCC3 in the DNA damage response, the two important reasons attracting our attention to the contribution of BRCC3 to NPC radioresistance is the high BRCC3 level in 3-year LRRFS and the overexpression of BRCC3 in radioresistant NPC cell lines. The BRCC3 level is upregulated in both inherent NPC cells (CNE1, HNE1) and radiation induced resistant CNE2R cells (Fig. 3). Additionally, the knockdown of BRCC3 expression sensitized the HNE1 and CNE2R cells to ionizing radiation (Fig. 4). This indicated that BRCC3 may be a potential therapeutic target for not just the treatment-naive patients, but for patients who suffered a relapse after standardized treatment.

Although a variety of studies revealed that the depletion of BRCC3 results in enhanced radiosensitivity in vitro, the mechanisms of the increased radiosensitivity caused by BRCC3 depletion have not yet been established and could be partially due to its interaction with BRCA1. BRCC3 directly interacts with BRCA1 at the region encompassing amino acids 502 to 1054 [12], which falls within the BRCA1 DNA-binding domain (amino acids 452-1079). BRCC3 deficiency inhibits BRCA1 focus formation and disrupts the IR-induced phosphorylation of BRCA1 [17] that may contribute to BRCA1-dependent DSB repair. Another mechanism lies in the BRCC3 de-ubiquitinating activity, which plays an important role for recruitment events in the repair of DSBs. BRCC3 disruption has been reported to be associated with increased 53BP1 and γH2AX foci accumulation after IR [15]. This is consistent with our result indicating that BRCC3 knockdown increased the persistence of γH2AX foci positive cells 24 h after irradiation (Fig. 4d,e), which indicated a delay of the DNA damage repair.

Regulation of the cell cycle may be another important mechanism for radiosensitization, as supported by our results that BRCC3 disruption increased cell-cycle arrest in nasopharyngeal carcinoma cell lines (Fig. 5a, b). The Cdk1-cyclin B complex is the main target molecule of the G2/M cell cycle checkpoint [36, 37]. The results of our study showed that BRCC3 knockdown upregulated cyclinB1 expression after irradiation compared to the control cells (Fig. 5c). In addition to BRCC3, the knockdown of other BRCC complex subunits, such as BRCA1, BRE, RAP80 and NBA1, also induced G2/M arrest [12, 38, 39], suggesting that the integrity of this complex is vital for the G2/M transition. However, the subunits may also play a separate role in the G2/M cell cycle regulation. Cdk1 binds with RAP80 protein and mediates its phosphorylation at an evolutionarily conserved Ser-677 residue, which is important for RAP80 functional sensitivity to IR and G2/M checkpoint control [38]. Nevertheless, further studies are needed to determine the exact mechanism for the G2/M arrest induced by BRCC3.

## Conclusion

This study demonstrates that BRCC3 overexpression, which contributes to a poorer prognosis in NPC patients, confers a stronger radiation resistance to NPC cells owing to more effective DNA damage repair and cell cycle regulation. Thus, effectively inhibiting BRCC3 expression is a potential therapeutic strategy for sensitizing resistant NPC cells to radiation.

## References

[1] Yu MC, Yuan JM (2002). Epidemiology of nasopharyngeal carcinoma. Semin Cancer Biol.

[2] Chan AT, Felip E (2009). Nasopharyngeal cancer: ESMO clinical recommendations for diagnosis, treatment and follow-up. Ann Oncol.

[3] Suarez C, Rodrigo JP, Rinaldo A, Langendijk JA, Shaha AR, Ferlito A (2010). Current treatment options for recurrent nasopharyngeal cancer. Eur Arch Otorhinolaryngol.

[4] Bekker-Jensen S, Lukas C, Kitagawa R, Melander F, Kastan MB, Bartek J (2006). Spatial organization of the mammalian genome surveillance machinery in response to DNA strand breaks. J Cell Biol.

[5] Rogakou EP, Boon C, Redon C, Bonner WM (1999). Megabase chromatin domains involved in DNA double-strand breaks in vivo. J Cell Biol.

[6] Lukas C, Falck J, Bartkova J, Bartek J, Lukas J (2003). Distinct spatiotemporal dynamics of mammalian checkpoint regulators induced by DNA damage. Nat Cell Biol.

[7] Huen MS, Grant R, Manke I, Minn K, Yu X, Yaffe MB (2007). RNF8 transduces the DNA-damage signal via histone ubiquitylation and checkpoint protein assembly. Cell.

[8] Kolas NK, Chapman JR, Nakada S, Ylanko J, Chahwan R, Sweeney FD (2007). Orchestration of the DNA-damage response by the RNF8 ubiquitin ligase. Science.

[9] Zhao GY, Sonoda E, Barber LJ, Oka H, Murakawa Y, Yamada K (2007). A critical role for the ubiquitin-conjugating enzyme Ubc13 in initiating homologous recombination. Mol Cell.

[10] Wang B, Elledge SJ (2007). Ubc13/Rnf8 ubiquitin ligases control foci formation of the Rap80/Abraxas/Brca1/Brcc36 complex in response to DNA damage. Proc Natl Acad Sci U S A.

[11] Mailand N, Bekker-Jensen S, Faustrup H, Melander F, Bartek J, Lukas C (2007). RNF8 ubiquitylates histones at DNA double-strand breaks and promotes assembly of repair proteins. Cell.

[12] Dong Y, Hakimi MA, Chen X, Kumaraswamy E, Cooch NS, Godwin AK (2003). Regulation of BRCC, a holoenzyme complex containing BRCA1 and BRCA2, by a signalosome-like subunit and its role in DNA repair. Mol Cell.

[13] Coleman KA, Greenberg RA (2011). The BRCA1-RAP80 complex regulates DNA repair mechanism utilization by restricting end resection. J Biol Chem.

[14] Davis AJ, Chi L, So S, Lee KJ, Mori E, Fattah K (2015). BRCA1 modulates the autophosphorylation status of DNA-PKcs in S phase of the cell cycle. Nucleic Acids Res.

[15] Shao G, Lilli DR, Patterson-Fortin J, Coleman KA, Morrissey DE, Greenberg RA (2009). The Rap80-BRCC36 de-ubiquitinating enzyme complex antagonizes RNF8-Ubc13-dependent ubiquitination events at DNA double strand breaks. Proc Natl Acad Sci U S A.

[16] Kato K, Nakajima K, Ui A, Muto-Terao Y, Ogiwara H, Nakada S (2014). Fine-tuning of DNA damage-dependent ubiquitination by OTUB2 supports the DNA repair pathway choice. Mol Cell.

[17] Chen X, Arciero CA, Wang C, Broccoli D, Godwin AK (2006). BRCC36 is essential for ionizing radiation-induced BRCA1 phosphorylation and nuclear foci formation. Cancer Res.

[18] Cao JY, Liu L, Chen SP, Zhang X, Mi YJ, Liu ZG (2010). Prognostic significance and therapeutic implications of centromere protein F expression in human nasopharyngeal carcinoma. Mol Cancer.

[19] Pan Y, Wang M, Bu X, Zuo Y, Wang S, Wang D (2013). Curcumin analogue T83 exhibits potent antitumor activity and induces radiosensitivity through inactivation of Jab1 in nasopharyngeal carcinoma. BMC Cancer.

[20] Qu C, Liang Z, Huang J, Zhao R, Su C, Wang S (2012). MiR-205 determines the radioresistance of human nasopharyngeal carcinoma by directly targeting PTEN. Cell Cycle.

[21] Song LB, Zeng MS, Liao WT, Zhang L, Mo HY, Liu WL (2006). Bmi-1 is a novel molecular marker of nasopharyngeal carcinoma progression and immortalizes primary human nasopharyngeal epithelial cells. Cancer Res.

[22] Dong JQ, Li MZ, Liu ZG, Zhong Q, Xiong D, Xu LH (2012). Establishment and characterization of a novel nasopharyngeal carcinoma cell line (SUNE2) from a Cantonese patient. Chin J Cancer.

[23] Deng R, Tang J, Ma JG, Chen SP, Xia LP, Zhou WJ (2011). PKB/Akt promotes DSB repair in cancer cells through upregulating Mre11 expression following ionizing radiation. Oncogene.

[24] van Vuurden DG, Hulleman E, Meijer OL, Wedekind LE, Kool M, Witt H (2011). PARP inhibition sensitizes childhood high grade glioma, medulloblastoma and ependymoma to radiation. Oncotarget.

[25] Kimple RJ, Smith MA, Blitzer GC, Torres AD, Martin JA, Yang RZ (2013). Enhanced radiation sensitivity in HPV-positive head and neck cancer. Cancer Res.

[26] Huang X, Taeb S, Jahangiri S, Emmenegger U, Tran E, Bruce J (2013). miRNA-95 mediates radioresistance in tumors by targeting the sphingolipid phosphatase SGPP1. Cancer Res.

[27] Y-f X, M-z L, Huang B, Chen J, Li Z, Wang H (2001). Cellular radiobiological characteristics of human nasopharyngeal carcinoma cell lines. Chin J Cancer.

[28] Pawlik TM, Keyomarsi K (2004). Role of cell cycle in mediating sensitivity to radiotherapy. Int J Radiat Oncol Biol Phys.

[29] Hartwell LH, Weinert TA (1989). Checkpoints: controls that ensure the order of cell cycle events. Science.

[30] Wang Z, Fan M, Candas D, Zhang TQ, Qin L, Eldridge A (2014). Cyclin B1/Cdk1 coordinates mitochondrial respiration for cell-cycle G2/M progression. Dev Cell.

[31] Chai KM, Wang CY, Liaw HJ, Fang KM, Yang CS, Tzeng SF (2014). Downregulation of BRCA1-BRCA2-containing complex subunit 3 sensitizes glioma cells to temozolomide. Oncotarget.

[32] Kauffmann A, Rosselli F, Lazar V, Winnepenninckx V, Mansuet-Lupo A, Dessen P (2008). High expression of DNA repair pathways is associated with metastasis in melanoma patients. Oncogene.

[33] Dyrskjot L, Thykjaer T, Kruhoffer M, Jensen JL, Marcussen N, Hamilton-Dutoit S (2003). Identifying distinct classes of bladder carcinoma using microarrays. Nat Genet.

[34] Veer LJ V’t, Dai H, van de Vijver MJ, He YD, Hart AA, Mao M (2002). Gene expression profiling predicts clinical outcome of breast cancer. Nature.

[35] Wang Y, Klijn JG, Zhang Y, Sieuwerts AM, Look MP, Yang F (2005). Gene-expression profiles to predict distant metastasis of lymph-node-negative primary breast cancer. Lancet.

[36] Smits VA, Medema RH (2001). Checking out the G (2)/M transition. Biochim Biophys Acta.

[37] Kim H, Chen J (2008). New players in the BRCA1-mediated DNA damage responsive pathway. Mol Cells.

[38] Cho HJ, Oh YJ, Han SH, Chung HJ, Kim CH, Lee NS (2013). Cdk1 protein-mediated phosphorylation of receptor-associated protein 80 (RAP80) serine 677 modulates DNA damage-induced G2/M checkpoint and cell survival. J Biol Chem.

[39] Wang B, Hurov K, Hofmann K, Elledge SJ (2009). NBA1, a new player in the Brca1 A complex, is required for DNA damage resistance and checkpoint control. Genes Dev.

